# Commonalities and Differences in NREM Parasomnias and Sleep-Related Epilepsy: Is There a Continuum Between the Two Conditions?

**DOI:** 10.3389/fneur.2020.600026

**Published:** 2020-12-11

**Authors:** Carlotta Mutti, Giorgia Bernabè, Noemi Barozzi, Rosario Ciliento, Irene Trippi, Giuseppe Pedrazzi, Nicoletta Azzi, Liborio Parrino

**Affiliations:** ^1^Department of Medicine and Surgery, Sleep Disorders Center, University of Parma, Parma, Italy; ^2^Unit of Neuroscience & Interdepartmental Center of Robust Statistics, Department of Medicine and Surgery, University of Parma, Parma, Italy

**Keywords:** disorders of arousal, parasomnia, sleep-related hypermotor epilepsy, SHE, cyclic alternating pattern, CAP, sleep microstructure

## Abstract

**Introduction:** Differential diagnosis between disorders of arousal (DoA) and sleep-related hypermotor epilepsy (SHE) often represents a clinical challenge. The two conditions may be indistinguishable from a semiological point of view and the scalp video-polysomnography is often uninformative. Both disorders are associated with variable hypermotor manifestations ranging from major events to fragments of a hierarchical continuum of increasing intensity, complexity, and duration. Given their semiological overlap we decided to explore the sleep texture of DoA and SHE seeking for similarities and differences.

**Methods:** We analyzed sleep macrostructure and CAP (cyclic alternating pattern) parameters in a cohort of 35 adult DoA patients, 40 SHE patients and 24 healthy sleepers, all recorded and scored in the same sleep laboratory. Nocturnal behavioral manifestations included minor motor events, paroxysmal arousals and major attacks in SHE, and simple, rising, or complex arousal movements in DoA.

**Results:** Compared to healthy controls, DoA and SHE showed similar amounts of sleep efficiency, light sleep, deep sleep, REM sleep, CAP subtypes. Both groups also showed slow wave sleep fragmentation and an increased representation of stage N3 in the second part of the night. The only discriminating elements between the two conditions regarded sleep length (more reduced in DoA) and sleep instability (more elevated in SHE). In DoA recordings, all motor episodes arose from NREM sleep: 37% during light NREM stages and 63% during stage N3 (simple arousal movements: 94%). In SHE recordings, 57% of major attacks occurred during stage N3.

**Conclusions:** So far, emphasis has been placed on the differentiation of sleep-related epilepsy and NREM arousal disorders. However, the impressive analogies between DoA and SHE suggest the existence of an underestimated continuum across the conditions, linked by increased levels of sleep instability, higher amounts of slow wave sleep and NREM/REM sleep imbalance. Sleep texture is extremely similar in the two conditions, although CAP metrics disclose quantitative differences. In particular, SHE patients show a higher arousal instability compared to DoA subjects. Given their clinical and epidemiological overlap, a common genetic background is also hypothesized. In such a perspective, we suggest that the consolidated dichotomy DoA vs. SHE should be reappraised.

## Introduction

Disorders of arousal (DoA) encompass heterogeneous motor behaviors (parasomnias) during non-REM sleep, e.g., sleepwalking, sleep terror, and confusional arousals, arising as a result of incomplete awakening (1).

DoA can be triggered by sleep deprivation, sleep disorders, medication, or psychosocial stressors and are accompanied by variable degrees of autonomic activation. Misperception, mental confusion, partial unresponsiveness to external stimuli, and retrograde amnesia are commonly reported during DoA episodes (2). Given its association with complex hypermotor behaviors and abnormal arousal reactions during sleep, the differential diagnosis between DoA and sleep-related hypermotor epilepsy (SHE) is often a challenging issue for clinicians. Both sleep disorders can either persist from childhood or appear *de novo* and may require different treatment strategies. DoA include a spectrum of disorders encompassing different motor manifestations with increasing semiological complexity which have been classified (3) as simple arousal movements (SAM), rising arousal movements (RAM) and complex arousal movements (CAM) and may occur in the same patient and in the same night. SHE itself is part of a spectrum ranging from minor motor events (MME), paroxysmal arousals (PA) and major attacks (4–8), often coexisting in the same patient and sometimes in the same night.

The clinical and polysomnographic differentiation between SHE, parasomnias and physiologic movements during sleep has always raised semiological difficulties and according to some authors a clearcut distinction cannot be carried out without video-polysomnographic analysis (v-PSG) (9). In contrast, Vignatelli et al. (10) describe major disagreement among sleep experts and trainees in distinguishing between PA and non-epileptic arousals. Other studies underlined the discriminatory informations provided by the sleep stage at onset, while ictal EEG features appear less useful (11).

DoA are traditionally considered as manifestations of fragmentary arousals occurring mostly during slow-wave sleep (SWS) (12): the mind is asleep, but the motor system is awake. Rhythmic movements within DoA are generally rare and may be seen either as part of exploratory movements in abortive sleepwalking or as part of complex DoA. In contrast, behaviors in SHE often display rhythmic components, including body rocking and rolling, bipedal cycling, and kicking. The differential diagnosis between DoA and SHE also relies on frequency and distribution pattern of the events: DoA episodes occur sporadically and less stereotyped in the first part of the night (dominated by deep NREM sleep) and are boosted by stage changes; SHE events are more frequent and highly stereotyped, prevail during light NREM stages, and may be preceded by abrupt arousal (8).

As behavioral events peculiar to DoA and SHE may occur in the same patient during the same night, differential diagnosis may become complicated, especially when only minor/mild events are available in the v-PSG recording. While major attacks in SHE are typically stereotyped and triggered by paroxysmal discharges, minor motor events may widely vary and appear similar to physiological movements during sleep. Furthermore, the association between minor motor events and epileptiform discharges is weak: it has been suggested that the former could reflect the activation of some innate motor patterns, not necessarily linked to paroxysmal events (7). In DoA patients, the awake EEG is normal, while PSG recordings often document the abrupt appearance of high-amplitude rhythmic slow waves before the episodes, followed by the persistence of either partial or complete sleep activity in the post-arousal recordings (1).

Brain activity during sleep is physiologically controlled by two driving forces: the sleep-promoting system and the arousal-promoting system, the latter connecting the sleeper with the surrounding world and crucial to restore wakefulness (13). This interplay is mirrored by the dynamic texture of sleep, which, within certain ranges, warrants flexible and adaptive strategies. Within NREM sleep stages, phasic EEG events are lumped in periodic clusters which define a cyclic alternating pattern (CAP). Recognized as the EEG biomarker of sleep instability, CAP is composed of a phase A of greater arousal (k-complexes, delta burst, polyphasic bursts, arousals) and a phase B of lesser arousal (baseline interval between consecutive A phases). While sleep stages and cycles are the expression of sleep macrostructure, CAP oscillations organized in sequences constitute sleep microstructure, which occurs either spontaneously or evoked by external stimuli and yields to consistent autonomic reactions (14). As the A phases of CAP encompass different transient events, they are classified as subtypes A1, A2, and A3, based on reciprocal proportion of high-voltage slow waves (EEG synchrony) and low-amplitude fast rhythms (EEG desynchrony). In the physiological architecture of sleep, subtypes A1 parallel the homeostatic process, while subtypes A2 and A3 are closely linked to the ultradian cyclicity. During the descending slope of sleep cycles CAP sequences preserve sleep against perturbations, boosting SWS, whereas during the ascending slope of the sleep cycle, microstructural fluctuations lighten sleep, and prepare the onset of REM periods. Therefore, CAP sequences can provoke both “arousal promoting” and “sleep promoting” reactions, depending on the background homeostatic pressure, and on the ongoing sleep stages (15, 16).

Previous studies showed a significant increase of CAP both in SHE (14) and in DoA patients (17) compared to healthy controls. Moreover, clinical events in both SHE and DoA are often triggered by an arousal event (phase A of CAP) indicating that SHE and DoA share common sleep features which can explain why it can become extremely difficult to distinguish the two conditions and why the same patient can present both seizures and parasomnias.

So far, particular emphasis has been placed on the differentiation of SHE and NREM arousal disorders (18). However, the two conditions share an impressive amount of common features. In particular, periodism, the attacks coinciding with the typical CAP recurrence (19), modulates both nocturnal epileptic (20) and parasomnic episodes (17, 21). In such a perspective, we suggest that the consolidated dichotomy DoA vs. SHE should be reappraised. To explore boundaries, gaps and overlaps in the two conditions, standard PSG measures, CAP parameters, and video findings of adult subjects with DoA were analyzed and compared with the data of age-balanced SHE patients and healthy controls recorded and scored in the same sleep laboratory.

## Materials and Methods

### Subjects

We reviewed the database of the Sleep Disorders Center at Parma University Hospital selecting patients with diagnosis of DoA who underwent nocturnal video-PSG in the time period between 2007 and 2019. A total of 234 patients were scrutinized, 199 patients were excluded for variable reasons including: coexistence of sleep disorders others than DoA, concomitant psychiatric or neurological conditions, incomplete follow-up or unavailability of video-PSG recording. We included all consecutive adult patients (≥ 18 years old) with at least 2 neurological visits and an overnight lab-setted v-PSG who received a diagnosis of DoA according to the ICSD criteria (International classification of Sleep Disorders, III Edition, American Academy of Sleep Medicine, 2014). A randomly selected group of patients with a clinical history of paroxysmal arousal, nocturnal wandering or hyperkinetic seizures, composed the SHE group (14), based on the diagnostic criteria established in 2016 (8). Eligible healthy controls were paid volunteers free of psychiatric, neurologic, and/or medical disorders, recruited through advertisement at the university hospital.

Exclusion criteria for all the subjects (patients and controls) were the following: (1) concomitant neurological, psychiatric or any other sleep disorders; (2) intake of medications known to influence sleep.

For each DoA patient we collected complete demographical and clinical data from medical recording and then anonymously abstracted them using a standardized data extraction spreadsheet. Specifically we recorded informations relative to age at onset, episodes frequency (divided in low: 1–2 per month; moderate: 1 per week; high: > 1 per week), previous personal medical history, family history for sleep disorders, with specific attention to NREM-parasomnia. Daytime sleepiness was assessed by means of the Epworth Sleepiness Scale (ESS): a score > 10 was considered clinically relevant. All enrolled patients carried out at least one lab-set full-night v-PSG recording.

The clinical, demographic and v-PSG features regarding SHE patients and healthy sleepers were collected from the Sleep Disorders Center at Parma University Hospital database. The major findings of epileptic patients and normal controls were published in a previous report (14). The study was regularly approved by the Local Ethics Committee with protocol number 9/2019/OSS^*^/AOUPR.

### PSG Evaluation

PSG recording was based on the international 10:20 system with 19 EEG channels on the scalp (Fp1, Fp2, F3, F4, F7, F8, C3, C4, P3, P4, O1, O2, T3, T4, T5, T6, FZ, CZ, PZ) referenced on mastoid, EOG for both eyes, EMG of the mentalis and limb muscles, ECG and synchronized audio-visual recording. A standard calibration of 50 mV/ mm with a time constant of 0.1 s and a high frequency filter in the 30 Hz range were applied. For all subjects bedtime was fixed at 10.30 pm. A resting wakefulness EEG of at least 20 min of duration was evaluated by a neurologist with expertise in epilepsy and sleep disorders. The detection of an apnea–hypopnea index >5/h and/or periodic limb movement (PLM) index >15/h of sleep represented exclusion criteria. Specifically “apneas” were defined as complete cessation of airflow for > 10 s, “hypopneas” were characterized by at least 30% drop in airflow from baseline value for at least 10 s and accompanied by either 3% reduction in SatO2% with respect to pre-event baseline and/or by an EEG arousal; “respiratory effort related arousal” were defined as sequences of breaths lasting at least 10 s and associated with increased respiratory efforts leading to arousal from sleep, not fulfilling diagnostic criteria for apnea nor hypopnea, in line with American Academy of Sleep Medicine (AASM) scoring rules (22).

#### Sleep Macrostructure

PSG scoring was carried out according to AASM rules (22). We measured sleep efficiency (SE), total sleep time (TST), stage 1 (N1) sleep time, stage 2 (N2) sleep time, stage 3 (N3) sleep time, REM sleep time.

#### Sleep Microstructure

CAP was performed following standardized guidelines (23) using Embla REM-logic software. For CAP analysis the following variables were evaluated: CAP rate (CAP time/total non-REM time × 100), CAP rate in NREM stages; CAP subtypes A1, subtypes A2, and subtypes A3.

### Classification of Nocturnal Motor Episodes

When DoA patients presented motor episodes during the recording night we described the clinical semiology and the sleep stage distribution. According to Loddo et al. (3), behavioral patterns of DoA patients were classified as: simple arousal movements (SAMs), rising arousal movements (RAMs) and complex arousal movements (CAMs). More specifically, SAMs referred to simple head movements comprehensive of head flexion/extension (SAM-A), with or without limb (SAM-B), or trunk minor motor activation (SAM-C). SAMs are far the commonest nocturnal motor manifestation in adults with DoA. RAMs are characterized by more complex in-bed motor patterns (trunk flexion or sitting) sometimes associated with speaking. Finally CAMs represent the most elaborate DoA motor pattern: patients with CAMs may leave their bed, manipulate objects, scream, or walk in the room.

Nocturnal epileptic seizures were classified as minor motor events, paroxysmal arousals and major events. Specifically minor motor events were represented by brief, simple and stereotyped movements involving either head, trunk or limbs; paroxysmal arousals were associated with sudden arousal and stereotyped motor activation with variable association of autonomic reactions and/or vocalization, lasting from 5 to 10 s and finally major events were the most complex episodes, consisting in complex stereotyped hypermotor patterns (including tonic–dystonic or hyperkinetic seizures and epileptic nocturnal wandering) with frequent autonomic activation, lasting on average 20–30 s (6). Only epileptic motor events supported by v-PSG evidence were taken into consideration.

## Statistical Analysis

Statistical analysis was performed using the open-source software Jamovi (24). All quantitative data were expressed as mean and standard deviation (SD). Qualitative data were reported as absolute frequency and percentage. A one-way ANOVA test assessed the differences among the mean values in the 3 groups (healthy controls, DoA, SHE). Normality of the data and homogeneity of variances were tested by the Shapiro-Wilk test and Levene's test, respectively. Categorical data were analyzed by the Pearson's chi-square test. Statistical significance was set at *p* < 0.05. Tukey *post-hoc* test was used to explore differences between groups after ANOVA. Effect size were also reported for ANOVA (Cohen's f and partial eta-squared) (25) and for Chi-square (Cramer's V) to measure the strength of the relationship between variables.

## Results

### Subjects

Due to strict inclusion criteria the final sample included 35 DoA subjects (12 female and 23 male, with a mean age of 28 ± 5 years). The SHE group included 40 subjects (20 male and 20 female; mean age: 31 ± 10 years). The control population encompassed 24 subjects (12 male and 12 female; mean age: 28 ± 7 years). The three groups showed similar age distribution (Table 1).

**Table 1:** Sleep macro and microstructure features in the three groups.

**Features**	***N***	**Group**	**Values (Mean +/– SD)**	***P*-value**	***post-hoc***	**Effect size**
Age (year)	24 35 40	HC DoA SHE	28 +/– 7 28 +/– 5 31 +/– 10	*p* = 0.187	HC vs. DoA: *p* = 0.950 HC vs. SHE: *p* = 0.327 DoA vs. SHE: *p* = 0.227	Overall effect size Cohen's *f* = 0.193 HC vs. DoA: *f* = 0.000 HC vs. SHE: *f* = 0.153 DoA vs. SHE: *f* = 0.171
						*F*_(2, 96)_ = 1.7063 Partial eta-square = 0.034
Sex (F/M)	24 35 40	HC DoA SHE	12/12 12/23 20/20	*p* = 0.323	HC vs. DoA: *p* = 0.228 HC vs. SHE: *p* = 1.000 DoA vs. SHE: *p* = 0.168	Phi, Cramer's V = 0.151 Cohen's w = 0.151
TST (min)	24 35 40	HC DoA SHE	466 +/ 24 389.92 +/– 56.97 438 +/– 74	***p*** **<** **0.001**	HC vs. DoA: *p* = 0.000 HC vs. SHE: *p* = 0.166 DoA vs. SHE: *p* = 0.0020	Overall effect size Cohen's *f* = 0.539
						HC vs. DoA: *f* = 0.518 HC vs. SHE: *f* = 0.196 DoA vs. SHE: *f* = 0.374
						*F*_(2, 96)_ = 12.728 Partial eta-square = 0.210
SE (%)	24 35 40	HC DoA SHE	93 +/– 5 87.59 +/– 11.59 85 +/– 17	*p* = 0.065	HC vs. DoA: *p* = 0.269 HC vs. SHE: *p* = 0.051 DoA vs. SHE: *p* = 0.667	Overall effect size Cohen's *f* = 0.255
						HC vs. DoA: *f* = 0.168 HC vs. SHE: *f* = 0.255 DoA vs. SHE: *f* = 0.092
						*F*_(2, 96)_ = 2.817 Partial eta-square = 0.055
N1 + N2 (%)	24 35 40	HC DoA SHE	55 +/– 14 55.03 +/– 8.6 53 +/– 12	*p* = 0.693	HC vs. DoA: *p* = 0.939 HC vs. SHE: *p* = 0.779 DoA vs. SHE: *p* = 0.726	Overall effect size Cohen's *f* = 0.084
						HC vs. DoA: *f* = 0.001 HC vs. SHE: *f* = 0.066 DoA vs. SHE: *f* = 0.075
						*F*_(2, 96)_ = 0.368 Partial eta-square = 0.007
N3 (%)	24 35 40	HC DoA SHE	20 +/– 13 26.44 +/– 8.55 28 +/– 10	***p*** **=** **0.011**	HC vs. DoA: *p* = 0.054 HC vs. SHE: *p* = 0.010 DoA vs. SHE: *p*= 0.792	Overall effect size Cohen's *f* = 0.299
						HC vs. DoA: *f*=0.229 HC vs. SHE: *f*=0.292 DoA vs. SHE: *f*=0.063
						*F*_(2, 96)_ = 4.705 Partial eta-square = 0.089
REM (%)	24 35 40	HC DoA SHE	25 +/– 5 17.02 +/– 5.2 19 +/– 6	***p*** **<** **0.001**	HC vs. DoA: *p* = 0.000 HC vs. SHE: *p* = 0.000 DoA vs. SHE: *p* = 0.269	Overall effect size Cohen's *f* = 0.570
						HC vs. DoA: *f* = 0.559 HC vs. SHE: *f* = 0.431 DoA vs. SHE: *f* = 0.159
						*F*_(2, 96)_ = 15.648 Partial eta-square = 0.246
CAP rate (%)	24 35 40	HC DoA SHE	32 +/– 5 50.86 +/– 10.24 72 +/– 11	***p*** **<** **0.001**	HC vs. DoA: *p* = 0.000 HC vs. SHE: *p* = 0.000 DoA vs. SHE: *p* = 0.000	Overall effect size Cohen's *f* = 1.733
						HC vs. DoA: *f* = 0.782 HC vs. SHE: *f* = 1.703 DoA vs. SHE: *f* = 1.004
						*F*_(2, 96)_ = 134.7 Partial eta-square = 0.737
CAP A1 (%)	24 35 40	HC DoA SHE	63 +/– 12 59.87 +/– 14.90 56 +/– 17	*p* = 0.193	HC vs. DoA: *p* = 0.722 HC vs. SHE: *p* = 0.180 DoA vs. SHE: *p* = 0.516	Overall effect size Cohen's *f* = 0.189
						HC vs. DoA: *f* = 0.080 HC vs. SHE: *f* = 0.184 DoA vs. SHE: *f* = 0.115
						*F*_(2, 96)_ = 1.6702 Partial eta-square = 0.034
CAP A2 (%)	24 35 40	HC DoA SHE	21 +/– 8 17.9 +/– 10.35 22 +/– 9	*p* = 0.154	HC vs. DoA: *p* = 0.421 HC vs. SHE: *p* = 0.909 DoA vs. SHE: *p* = 0.142	Overall effect size Cohen's *f* = 0.199
						HC vs. DoA: *f* = 0.128 HC vs SHE: *f* = 0.043 DoA vs SHE: *f* = 0.194
						F_(2, 96)_ = 1.9086 Partial eta-square = 0.038
CAP A3 (%)	24 35 40	HC DoA SHE	16 +/– 6 22.25 +/. 13.25 22 +/– 10	*p* = 0.052	HC vs. DoA: *p* = 0.071 HC vs. SHE: *p* = 0.076 DoA vs. SHE: *p* = 0.994	Overall effect size Cohen's *f* = 0.257
						HC vs. DoA: *f* = 0.233 HC vs. SHE: *f* = 0.229 DoA vs. SHE: *f* = 0.011
						*F*_(2, 96)_ = 3.0576 Partial eta-square = 0.060

*DoA, disorder of arousal; SHE, sleep-related hypermotor epilepsy; HC, healthy controls; TST, total sleep time; SE, sleep efficiency. Significant results are marked in bold*.

*Continuous variables: p-value from one-way Anova with Tukey HSD post-hoc test*.

*Categorical data: p-value from Pearson's Chi-square test with likelihood-ratio test*.

*Effect size: according to Jacob Cohen f values of 0.10, 0.25, and 0.40 represent small, medium, and large effect sizes, respectively; partial eta squared is a measure of the proportion of the total variance in a dependent variable that is associated with the membership to different groups. Values of eta-squared of 0.01, 0.06, and 0.14 represent small, medium, and large effect sizes, respectively*.

#### DoA Group

A family history for sleep disorders was documented in 6 cases (17%), disease onset during childhood was reported by 22 patients (63%), while the other 13 subjects (37%) developed NREM sleep parasomnia after the age of 18. Five patients (14%) presented high frequency of DoA episodes (>1 per week), 15 patients (43%) reported usually one episode per week and the remaining 15 patients (43%) had a mean of one or two episodes per month. Five patients (14%) suffered from concomitant psychiatric diseases including depression, anxiety and panic attacks. Daytime sleepiness (measured by ESS, cut-off >10) was found in 9 patients (23%).

#### SHE Group

In most patients with SHE, nocturnal motor events were already present at childhood. One patient suffered from mild perinatal hypoxia, two presented febrile convulsion during childhood and one had minor head trauma. No patient reported a family history of epilepsy, but 10 patients described parasomnias in first-degree relatives. Diurnal seizures were never reported and no patient showed coexisting neurologic, psychiatric or medical disorders known to affect sleep architecture. All SHE patients complained of excessive daytime sleepiness (ESS: 16 ± 4). Wakefulness EEG showed focal epileptiform abnormalities only in a minority of cases (5/40, 12.5%). Semiology of nocturnal episodes included paroxysmal arousals, focal tonic-dystonic seizure, hyperkinetic seizure, or prolonged motor behavior including epileptic nocturnal wandering. Brain MRI was unremarkable in all SHE patients. According to 2017 ILAE classification (26) all included subjects were classified as affected by focal epilepsy of unknown etiology.

### Sleep Macrostructure

The standard sleep measures in the three groups are detailed in Table 1. One-way ANOVA highlighted significant differences between groups with respect to TST, N3 and REM representation. Tukey *post-hoc* test revealed that, compared to healthy controls, TST was reduced significantly in DoA (−76 min, *p* = 0.0001) but not significantly in SHE (−28 min, *p* = 0.166). TST was also significantly lower in DoA compared to SHE (−48 min, *p* = 0.002). Overall, SE was not modified (*p* = 0.065) but both DoA (88%) and SHE (85%) presented values <90%.

The three groups showed similar amounts of light sleep (N1 + N2). Overall stage N3 was significantly different between the three groups (*p* = 0.011), being enhanced in DOA (+6%, *p* = 0.054) and in SHE (+8%, *p* = 0.010) with respect to healthy sleepers but no difference was found between the two clinical conditions (*p* = 0.792). Compared to healthy sleepers, REM sleep was reduced (*p* < 001) in DoA (−8%, *p* = 0.0001) and in SHE (−6%, *p* = 0.0001) with similar values in both conditions (*p* = 0.269).

In the 9 DoA patients complaining of excessive daytime sleepiness compared to the remaining 26 DoA subjects with an ESS ≤ 10, higher percentages of N3 (33 ± 9 vs. 24 ± 7; p = 0.004) and lower amounts of REM sleep (13% ± 5 vs. 18% ± 5; p = 0.014) were found.

### Sleep Microstructure

Tables 1, 2 showed the mean microstructural data in the three groups. Overall one-way ANOVA described significant differences between the three groups with respect to CAP rate, CAP rate in N2 and CAP rate in N3.

**Table 2:** CAP rate during stage N2 and N3 of NREM sleep in the three groups.

**Features**	**Group**	**Values (Mean +/– SD)**	***P*-value**	***post-hoc***	**Effect size**
CAP rate SWS (%)	HC DoA SHE	30 +/– 8 65,9 +/– 17,3 76 +/−24	***p*** **<** **0.001**	HC vs. DoA: *p* = 0.000 HC vs. SHE: *p* =0.000 DoA vs. SHE: *p* = 0.0792	Overall effect size Cohen's *f* = 10,607
					HC vs. DoA: *f* = 0.7624 HC vs. SHE: *f* = 1.0440 DoA vs. SHE: *f* = 0.2427
					*F*_(2, 90)_ = 45.618 eta squared = 0.503
CAP rate N2 (%)	HC DoA SHE	34 +/– 6 50,1 +/– 14,7 55 +/– 14	**p** **<** **0.001**	HC vs. DoA: *p* = 0.000 HC vs. SHE: *p* = 0.000 DoA vs. SHE: *p* = 0.2588	Overall effect size Cohen's *f* = 0.6993
					HC vs. DoA: *f* = 0.4951 HC vs. SHE: *f* = 0.6902 DoA vs. SHE: *f* = 0.1705
					F_(2, 90)_ = 21.045 eta squared = 0.319

*DoA, disorder of arousal; SHE, sleep-related hypermotor epilepsy; HC, healthy controls; SWS, slow wave sleep. Significant results are marked in bold P-values from one-way Anova with Tukey HSD post-hoc test. Effect size: according to Jacob Cohen f values of 0.10, 0.25, and 0.40 represent small, medium, and large effect sizes, respectively; partial eta squared is a measure of the proportion of the total variance in a dependent variable that is associated with the membership to different groups. Values of eta-squared of 0.01, 0.06, and 0.14 represent small, medium, and large effect sizes, respectively*.

In details, compared to healthy controls, CAP rate values were higher (*p* <0.001) in DoA (+ 19%) and even more elevated in SHE (+ 40%), with significant differences between the two conditions (*p* = 0.0001). In the three groups, CAP subtypes maintained the physiological ranking A1 > A3 > A2, with a similar increased representation of phases A3 (*p* = 0.052) in DoA (+6%, p = 0.071) and SHE (+6%, *p* = 0.076), presenting trends toward significancy.

CAP rate values in stages N2 and N3 were significantly higher in both DoA and SHE compared to healthy sleepers (*p* < 0.001) (details in Table 2).

### EEG Features

Epileptiform discharges during wakefulness occurred only in 5 SHE patients. During sleep, EEG abnormalities were identified in 28 of the 35 DoA patients (80%) and in all the SHE patients, the vast majority being represented by focal spikes. Comparing DoA patients with EEG abnormalities vs. DoA patients showing normal EEG no significant differences were detected with respect to sleep macrostructural and microstructural features: TST (*p* = 0,140), SE (*p* = 0,367), N1 + N2% (*p* = 0,297), N3% (*p* = 0,728), REM% (*p* = 0,080), CAP rate (*p* = 0,911), CAP subtype A1 (*p* = 0,677), A2 (*p* = 1,000), A3 (*p* = 0,676).

### Classification of Nocturnal Motor Episodes

#### DoA Group

**Figure 1:**
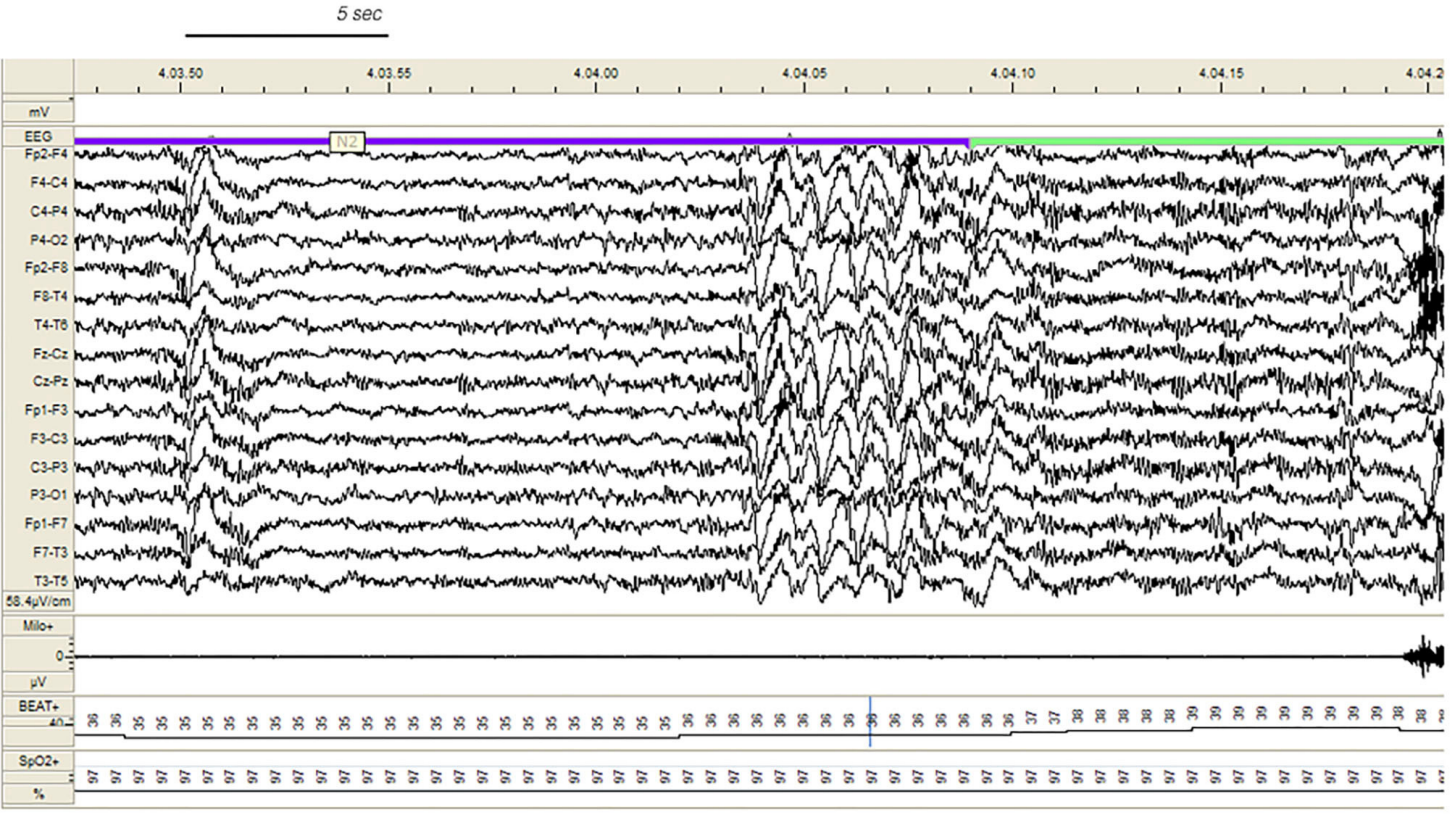
Confusional arousal beginning during stage N2 in a DoA patient. A high amplitude EEG burst of spiky slow waves precedes the episode. Notice the coexistence of autonomic arousal (heart rate acceleration) and the absence of respiratory events; EEG sensitivity 58.4 mV/cm; Milo+, chin electromyography; BEAT, heart frequency; SpO2+, oxygen saturation.

During v-PSG registration at least one DoA episode occurred in 27 patients (77%). All episodes were recorded during NREM sleep and in most cases arised from a phase A of CAP (Figure 1): 37 % occurred during light NREM sleep (stages N1 and N2) and 63% during N3. DoA episodes included episodes of confusional arousals, sleep terrors, sleep-walking, vocalizations (moaning or mumbling), laughing, simple movements (face or nose touching), eye opening, oro-masticatory movements. A total of 48 episodes were recorded: they were classified as simple arousal movements (SAM) (45 episodes), rising arousal movements (RAM) (2 episodes) or complex arousal movements (CAM) (1 episode). The SAM group included 9 SAM-A (20%), 23 SAM-B (51%) and 13 SAM-C (29%) patterns.

#### SHE Group

**Figure 2:**
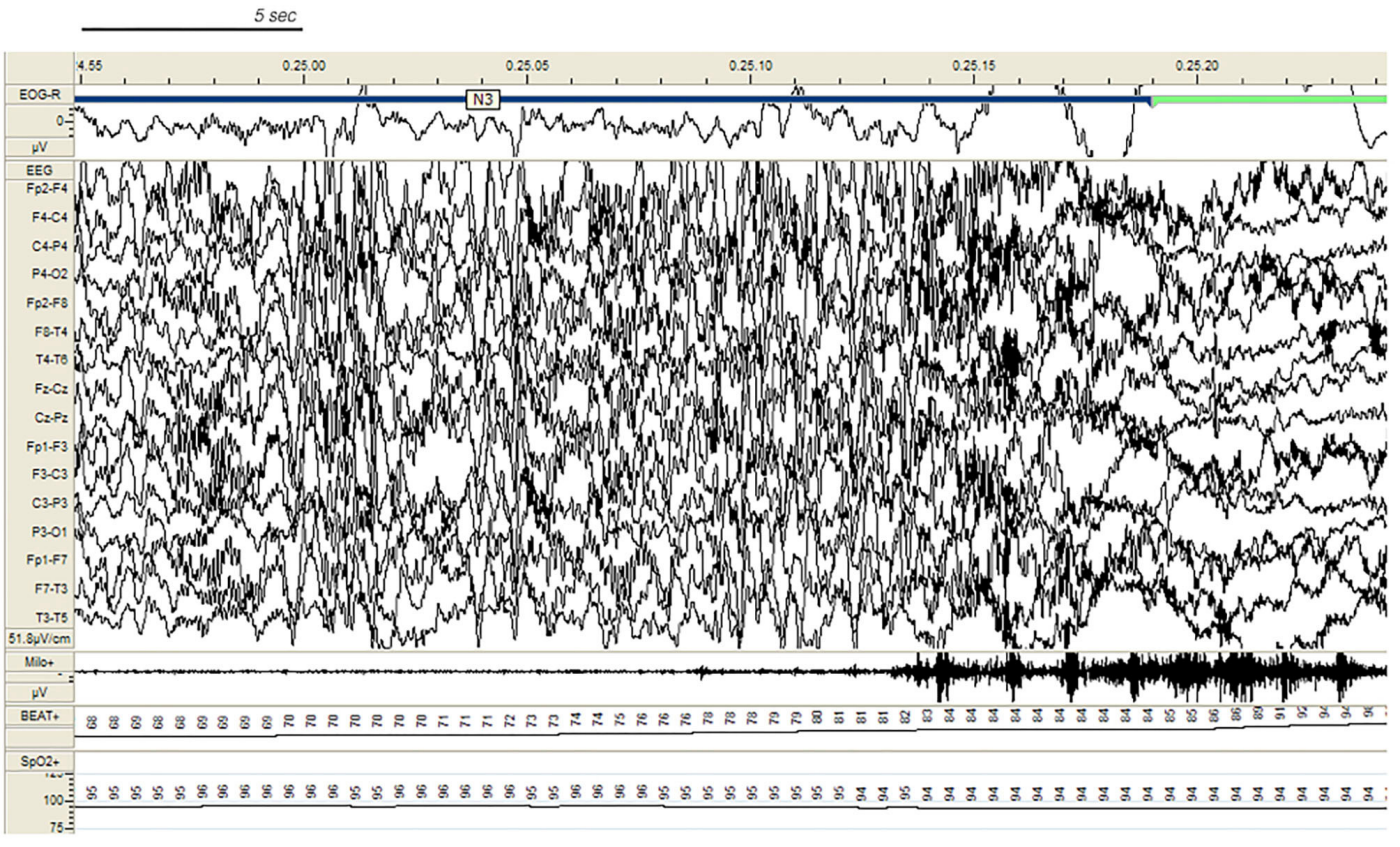
Paroxysmal arousal in a patient with SHE beginning during stage N3. Bursts of generalized high amplitude epileptiform discharges can be appreciated. Autonomic activation coexists. EEG sensitivity 51.8 mV/cm; Milo+: chin electromyography; BEAT, heart frequency; SpO2+, oxygen saturation.

In the SHE group, 33 patients (83%) reported multiple nocturnal episodes every night, whereas self-reported or witnessed seizures recurred weekly in 7 patients. Approximately 60% of the total amount of NREM sleep seizures arose from N2, but most major events (57%) showed a preferential occurrence during SWS (Figure 2). Ninety percent of total NREM seizures occurred during a CAP sequence, and CAP-related seizures always occurred in association with a phase A.

## Discussion

Both DoA and SHE presented impressive overlaps of sleep macro- and microstructural parameters. Compared to healthy controls, the two sleep disorders showed similar amounts of sleep efficiency, light sleep (N1+N2), deep sleep (N3), REM sleep, CAP subtypes (A1, A2, A3). Both groups also showed SWS fragmentation and an increased representation of stage N3 in the second part of the night. The only discriminating elements between the two conditions regarded sleep length (TST more reduced in DoA) and sleep instability (CAP rate more elevated in SHE).

### Sleep Texture

**Figure 3:**
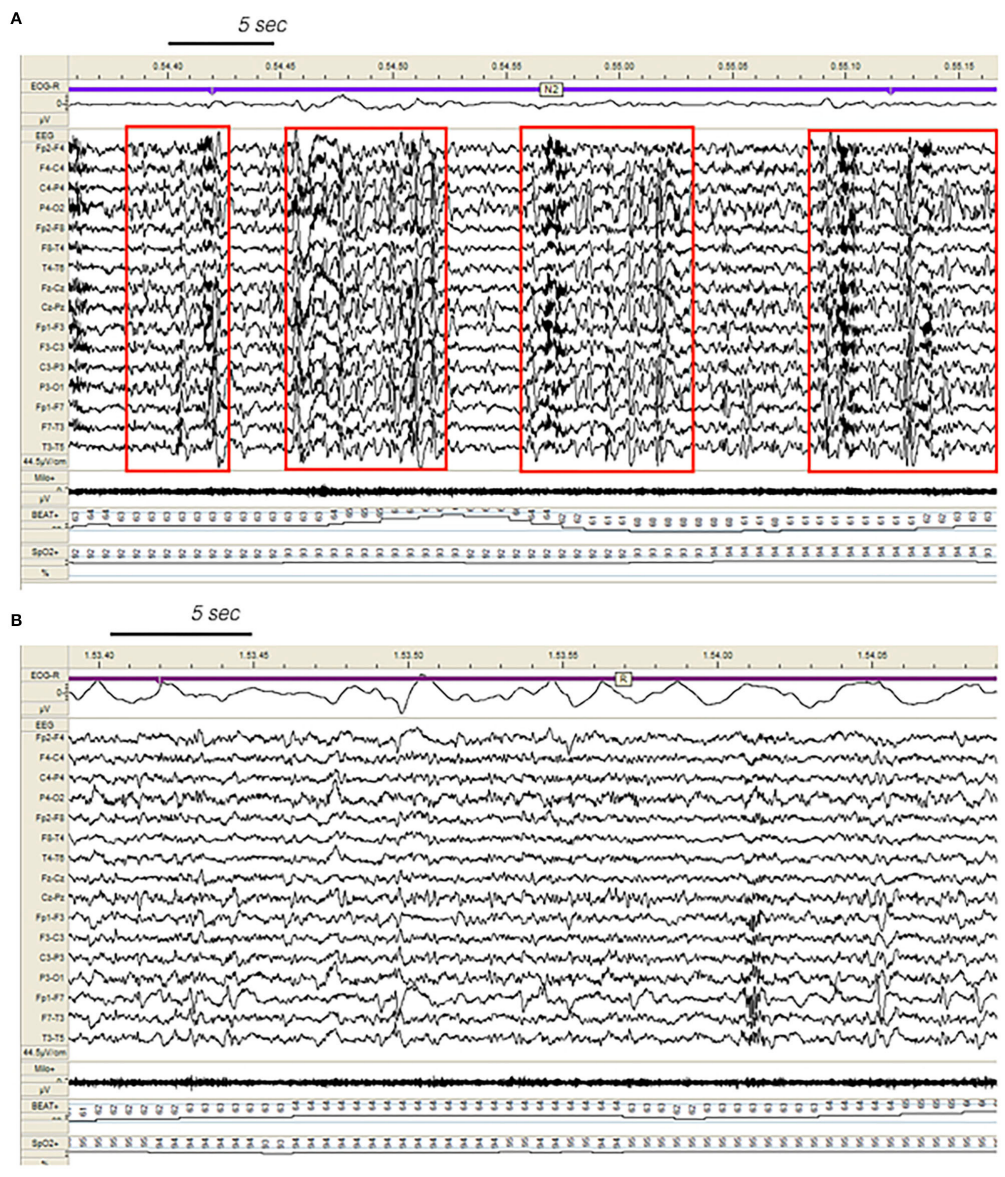
Dynamics of epileptiform discharges during NREM and REM sleep in a patient with SHE. **(A)** Bursts of generalized epileptiform discharges enhancing cyclic alternating pattern (CAP) fluctuations during stage N2 in a patient with SHE. Red boxes highlight CAP cycles. Transient heart rate fluctuations consistent with sleep instability. **(B)** REM sleep constrains epileptic phenomena and may provide information on the localization of seizure onset zone (left focal fronto-temporal spikes and sharp-waves). EEG sensitivity 44.5 mV/cm; Milo+, chin electromyography; BEAT, heart frequency; SpO2+, oxygen saturation.

The PSG alterations of DoA and SHE seem to be peculiar to the conditions. Indeed other sleep disorders including insomnia, periodic limb movements, sleep disordered breathing may present reduced sleep efficiency and increased sleep instability, but a significant increase of stage N3 is a really uncommon finding. In our DoA sample, the excess of SWS probably accounted for the lack of sleepiness in most patients (77%). On the contrary, in the SHE group, the protective action of N3 on daytime vigilance was counteracted by an excessive amount of CAP rate, a magnitude already described in severe obstructive sleep apnea (27–29), which is often accompanied by daytime sleepiness. The supplement of unstable sleep in the SHE group is fueled by a number of disturbing factors including ictal and interictal EEG paroxysms acting as noise equivalents on the neural circuitry (Figure 3). In a study on SHE patients (30) treated with antiepileptic agents, CAP rate dropped from 71% (no medication) to 59% (medication), remaining widely above the expected physiological value of 33% (healthy controls). Effective treatment reduced the total amount of sleep seizures of approximately 25%, but the persistence of EEG discharges fanned arousal oscillations during NREM sleep, producing an increase of reactive A phases and therefore boosting high CAP rate values. It is interesting to notice that medication in SHE patients was associated with an additional growth of stage N3 (+3%), which was impressively high (28%) even without medication.

### Trait vs. State Features

DoA and SHE share a number of specific EEG trait-markers and state-markers embedded within their sleep texture. Whether the elevated percentages of N3 are an intrinsic feature (trait) of both SHE and DoA or the compensatory by-product of a non-consolidated SWS due to motor events and/or EEG paroxysms occurring in NREM sleep (state) remains an open question. Probably, both assumptions stem from a common root. In DoA patients, impairment of sleep intensity and depth (31, 32) determines vulnerable and discontinuous slow waves during stage N3 (33). DoA sleep recordings are also characterized by an excessive fragmentation of SWS independent of concomitant parasomniac behaviors (34). Moreover, patients with DoA suffer from more frequent and longer arousals and awakenings from N3 than controls (35). Factors interfering with the build-up and maintenance of SWS, such as an excess and/or an abnormal distribution of CAP could also play a role in the pathophysiology of the disease. Accordingly, both SHE and DoA recordings were characterized by high amounts of SWS in the second half of the night: probably the result of an adaptive intra-night homeostatic recovery of stage N3 due to disturbed and inadequate SWS consolidation occurring in the initial sleep cycles.

### Triggers

**Figure 4:**
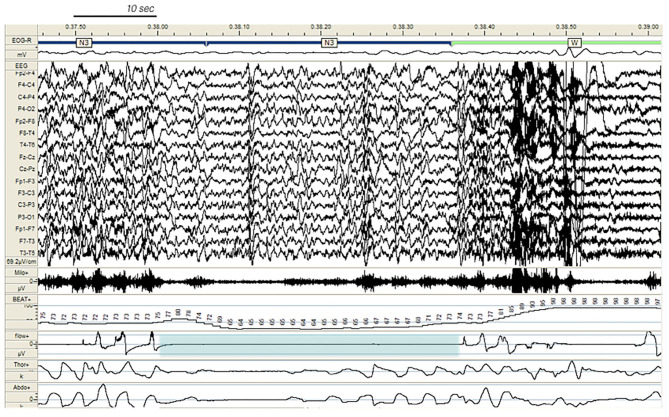
Paroxysmal arousal in a patient with SHE and untreated obstructive sleep apnea (not included in the study), beginning during stage N3 and triggered by a respiratory event (blue shadow). Bursts of generalized epileptiform discharges, slightly prevailing over the left hemisphere, which can be appreciated throughout the recording, are increased in amplitude during arousals. EEG sensitivity 69.2 mV/cm; Milo +, chin electromyography; BEAT, heart frequency; flow+, airflow thermistor; Thor+, plethysn1ography thoracic band; Abdo+, plethysmography abdominal band.

In our study, 80% of DoA subjects and all SHE patients presented EEG abnormalities during sleep, a perturbing factor which can trigger a phase A of CAP and subsequent arousal instability. In addition, a number of sleep disorders can also promote the unstable NREM sleep background on which DoA and SHE events occur. The triggering role of sleep deprivation (2, 36), medications; (37, 38), and sleep disordered breathing (38), (see Figure 4), including upper airway resistance syndrome (21) should be systematically searched for and treated in the presence of parasomnias or sleep-related epileptic episodes. Some authors (12, 39) have considered hypersynchronous slow delta activity (HSDA), i.e., bursts of high amplitude slow delta with fronto-central gradient occurring during NREM sleep, as the typical triggering EEG pattern of DoA (Figure 1). However, HSDA presents a clear overlap with CAP subtype A1 (21) and the unstable background may lead to stage N3 vulnerability which promotes the occurrence of motor episodes. According to our results both DoA and SHE patients presented significantly higher levels of SWS instability with respect to healthy sleepers (CAP in N3 being, respectively, +36 and + 46%). Similar results were described in a cohort of adult DoA patients and designated as “SWS fragmentation index” (34). In this dynamic landscape, subtypes A3 (21), which are the longest A phases of CAP (40) and are increased in sleepwalkers (21), probably play a weakening effect on N3 consolidation and are involved in the typical sleep-state dissociation when SWS and arousals coexist.

### NREM/REM Imbalance

Our SHE and DoA recordings were characterized by a significant reduction of REM sleep suggesting an imbalanced control of REM-on and REM-off forces. REM and NREM sleep are mutually linked and regulated by a reciprocal interaction (41). REM sleep also attenuated the spreading of EEG paroxysms, acting as a protective stage towards propagation of epileptiform discharges (42) (Figure 3).

Neurophysiological and neuroimaging studies in subjects with DoA have provided evidence of abnormal brain functioning not only during SWS but also during REM sleep (43). Quantitative EEG analysis carried out in sleepwalkers during non-sleepwalking nights shows that the absolute power of delta waves is significantly lower in sleepwalkers compared to controls during the first NREM-REM cycle (*p* = 0.03) and a very important trend (*p* = 0.059) is noted for the second sleep cycle (21). REM sleep is frequently curtailed also in adults (44) and children (45) suffering from sleep-related epilepsy. When antiepileptic medication attenuates the occurrence of major episodes in SHE patients, sleep cycles recover a physiological architecture and a normal REM-latency due to a more solid sleep structure especially in the first part of the night (30). In contrast, the A3 phases, which are also physiologically involved in the ultradian process of sleep (46) and show increased amounts in both untreated SHE (14) and DoA (21, 34), are unaffected by antiepileptic therapy (30).

### Clinical Manifestations: The Role of Sleep Staging and Arousal Instability

In line with previous reports, the commonest motor manifestation in our DoA group was represented by SAM (94%). A v-PSG assessment of 334 DoA episodes documented that 84% were SAM, 10% were RAM and 5% were CAM (47). In SHE patients, the majority of NREM seizures arose from stage N2, but most major attacks showed a preferential occurrence during SWS (57%). The different distribution of motor episodes across the night and within the NREM stages is a widely accepted issue (6, 35, 48). According to a recent study, the occurrence of at least one minor event during stage N3 is highly suggestive for DoA, while the occurrence of at least one major event outside stage N3 is highly suggestive for SHE (47). However, in the same study, the number of major events in stage N3 per subject coincided in both DoA and SHE patients (47), suggesting that sleep staging is not a major element for the differential diagnosis (8).

Probably, a different modulation of NREM stages on major and minor motor events in DoA and SHE patients is a more plausible statement. A close relation between MME and arousal fluctuations is also a consolidated issue (20). Therefore, besides classifying pathologies (DoA and SHE) according to the stage-distribution of nocturnal episodes, perhaps a greater attention on the unstable balance between arousal-promoting and sleep-promoting forces may provide additional informations regardless of the ongoing sleep stage.

### Central Pattern Generators (CPG)

**Figure 5:**
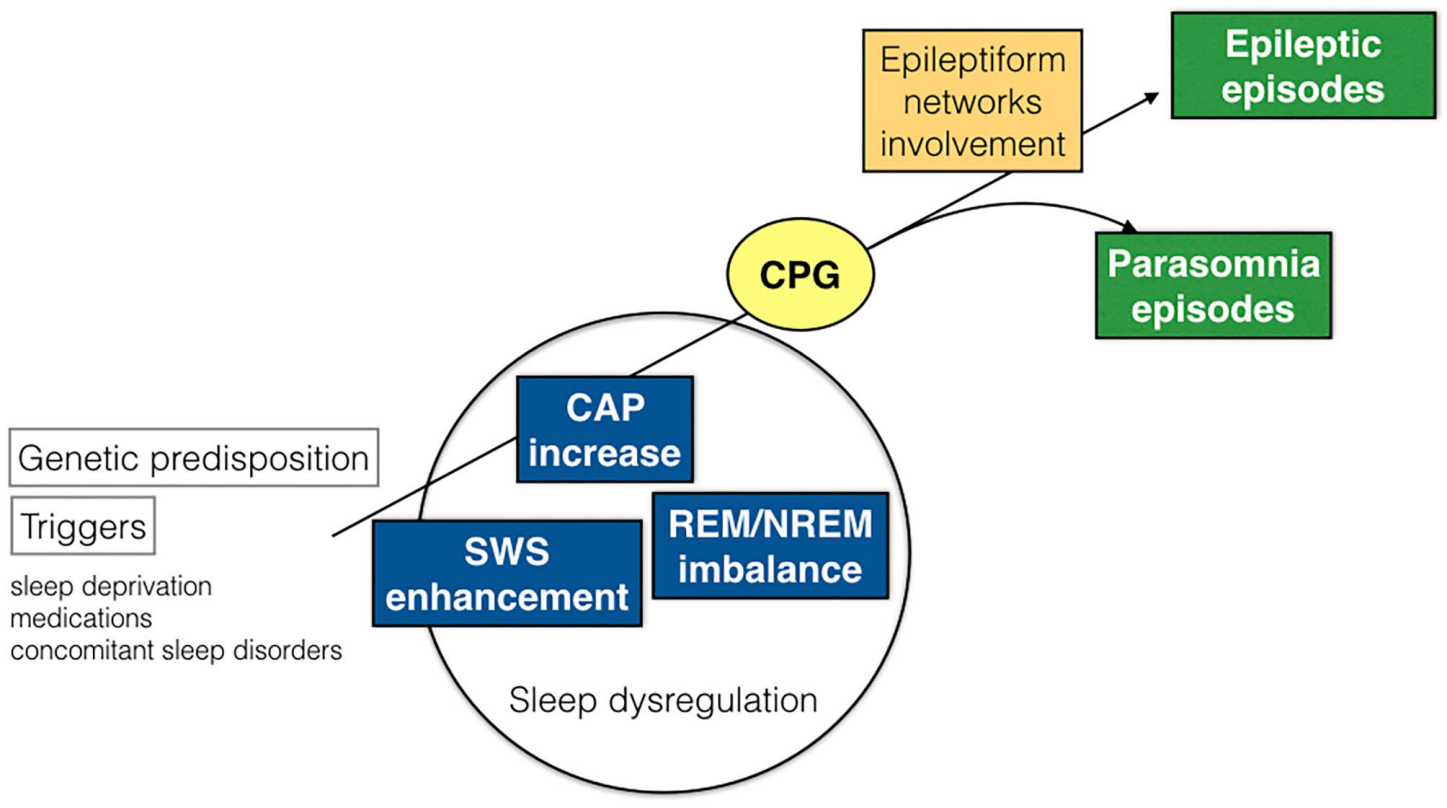
Schematic representation of pathogenetic commonalities between DoA and SHE. CAP, cyclic alternating pattern; SWS, slow wave sleep; CPG, central pattern generators.

As the great majority of nocturnal episodes were simple arousal movements (94% in the DoA group) or minor motor events (75% in the SHE group), the recorded behaviors probably expressed the fragments of a hierarchical continuum characterized by increasing intensity, complexity, and duration of the nocturnal episodes. The motor patterns which are already written in the brain codes need a window of arousal to become visibly apparent (40). Encoded CPG seem to be involved in the genesis of involuntary movements during sleep (49). The CPG system is composed of spinal and brainstem networks regulated by a supraspinal circuitry and produces coordinated and stereotyped locomotor movements such as walking or swimming, important for survival (50). The repetitive arousals of CAP activate these cortico-subcortical-spinal pathways, facilitating or releasing sleep-related behaviors such as NREM parasomnia or seizures. Regardless of the sleep disturbance, the arousal-induced activation of the CPG system generates stereotyped motor manifestations that often cause difficulties in the differential diagnosis between NREM parasomnias and SHE (Figure 5). It must be recalled that even highly stereotyped minor motor events can occur in the absence of an epileptiform discharge (7). In other words, CAP can be a common denominator of an arousal-related motor dishinibition whether or not epileptic in origin (51). The behavioral outcome relies on a number of factors including the local cerebral regions and activated networks.

### NREM vs. REM Parasomnias

These findings corroborate the impressive overlap between DoA and SHE in terms of semiology, EEG features, sleep patterns, cerebral regions and common triggers. Even if we are dealing with two distinct conditions, the blurred boundaries between them support the possibility of a continuum between DoA and SHE. The imbalance between arousal and sleep forces may entail variable motor manifestations determined by multiple factors, i.e., genetic predisposition, involvement and thresholds of specific brain areas and neural circuits, CPG, paroxysmal discharges, and activation of epileptic networks. However, the numerous commonalities suggest that DoA and SHE share basic NREM sleep-related pathogenetic mechanisms. Accordingly, the high amounts of CAP rate found in both DoA and SHE patients are opposed to the reduced levels of CAP rate in REM sleep parasomnias (52).

### Arousal Models Commonalities for DoA and SHE

So far three fascinating pathophysiological hypothesis focusing on arousal system functioning have been formulated to explain commonalities between SHE and DoA, respectively, named the “liberation,” “dissociation,” and “pathological” arousal models (53). The first focuses on functional de-inactivation of frontal lobe by subcortical nuclei (CPG) due to variable external or internal stimuli (54); the second recognizes the simultaneous mixed sleep and awake state existence as a major determinant for clinical manifestations (55, 56); the third harmonizes the previous models, assuming the existence of a gain-of-function of frontal cortical acetilcholine receptors in both SHE and DoA, explaining their semiological differences according to underlying facilitatory circumstances (53).

### Limitations and Unanswered Questions

Despite these consistent clues, a number of questions need to be addressed. Is the strong convergence between DoA and SHE basically due to our clinical difficulty to distinguish single sleep disorders? Are we using adequate tools to investigate behavioral manifestations during sleep? Are we dealing with a two-faced entity, which can offer one of the two sides of the coin even in the same patient and in the same night? Does it really matter to establish that a nocturnal motor event occurs in stage N2 or stage N3 if the common background is an unstable NREM sleep? Why are antiepileptic drugs often effective in the treatment of DoA episodes? Challenging issues, which represent intrinsic weaknesses of the study but encourage further investigations on the distinctive features and common nature of DoA and SHE. Another limitation of our investigation could be attributed to its retrospective nature, to the relatively small sample and to partial exploitation of published data (14). However, the entire framework–recruitment and recording–was carried out in a homogeneous setting (Sleep Disorders Center at Parma University Hospital) and scoring was completed by the same sleep team. Finally our SHE cohort included 10/40 patients with a positive family history for NREM sleep parasomnia in their first-degree members. Even if none of the included SHE patients presented nocturnal DoA-like manifestations nor other known sleep disorders, previous studies demonstrated (6, 57) that epileptic and parasomnic events frequently coexist and their semiological differentiation, especially when minor motor episodes prevail, is often a challenging issue (10) which requires accurate evaluation (3).

### Conclusions and Perspectives

SHE is considered a rare disease, with a crude prevalence among adults around 1.8/100.000 (58), while the prevalence rate of sleepwalking is estimated 5% in children and 1.5% in adults (59). As a possible explanation of these diverging data, SHE represents the tip of the iceberg acting in the same pathophysiological continuum of DoA, with potential underestimation due to the numerous diagnostic issues described in the present study. A common genetic background shared by DoA and SHE is also hypothesized. Familial aggregation of patients with diagnosed SHE and the higher frequency of arousal parasomnias in SHE probands and their relatives compared with a control population (18, 57, 60) support an intriguing affinity. The involvement of cholinergic pathways has been suggested in abnormal arousal reaction (57, 61). In particular, acetilcholine is one the major neurotransmitters of the ascending reticular activating system and nicotinic acetylcholine receptors are widely distributed in the brain and modulate arousal oscillations at cortical and subcortical levels (53, 62). Given the clinical and electrophysiological commonalities between SHE and DoA and their frequent overlap in the same patients and families, further research on the cholinergic system and other neurotransmitters involved in the modulation of arousal and sleep is a mandatory challenge (63).

Recently, a provocative paper highlighted the impressive parallelism between DoA and SHE based on the arousal system's hyperfunction and NREM sleep dissociation states (64). A brilliant conclusion on the dual nature of DoA and SHE is also available in The Philosophy of Sleep written almost 200 years ago. The author describes “the case of a watchmaker's apprentice who had an attack of sleep-walking every fortnight. In this state, though insensible to all external impressions, he would perform his work with his usual accuracy, and was always astonished, on awaking, at the progress he had made. The paroxysm began with a sense of heat in the epigastrium extending to the head, followed by confusion of ideas and complete insensibility, the eyes remaining open with a fixed and vacant stare. This case, which undoubtedly originated in some diseased state of the brain, terminated in epilepsy” (65).

## Data Availability Statement

The raw data supporting the conclusions of this article will be made available by the authors, under request.

## Ethics Statement

The studies involving human participants were reviewed and approved by Comitato Etico dell'area Vasta Emilia Nord (AVEN). The patients/participants provided their written informed consent to participate in this study.

## Author Contributions

GB, NB, IT, NA and RC collected the data. GP made statical analysis. CM and LP drafted the manuscript. All authors contributed to the design of the study protocol, critically reviewed the manuscript, and approved its submitted version.

## Conflict of Interest

The authors declare that the research was conducted in the absence of any commercial or financial relationships that could be construed as a potential conflict of interest.
